# DNA damage-induced YTHDC1 O-GlcNAcylation promotes homologous recombination by enhancing m^6^A binding

**DOI:** 10.1016/j.fmre.2023.04.017

**Published:** 2023-06-05

**Authors:** Mengyao Li, Jie Li, Yibo Wang, Jianxin Zhao, Aiyun Yuan, Weidong Dong, Linlin Kong, Suwei Dong, Weijie Qin, Yun-Gui Yang, Xiaohui Wang, Chen Wu, Jing Li

**Affiliations:** a College of Life Sciences, Institute of Life Sciences and Green Development, Hebei University, Baoding 071002, China; b Beijing Key Laboratory of DNA Damage Response, College of Life Sciences, Capital Normal University, Beijing 100048, China; c Laboratory of Chemical Biology, Changchun Institute of Applied Chemistry, Chinese Academy of Sciences, Changchun 130022, China; d State Key Laboratory of Natural and Biomimetic Drugs, Chemical Biology Center, and School of Pharmaceutical Sciences, Peking University, Beijing 100191, China; e State Key Laboratory of Proteomics, National Center for Protein Sciences – Beijing, Beijing Proteome Research Center, Beijing Institute of Lifeomics, Beijing 102206, China; f CAS Key Laboratory of Genomic and Precision Medicine, Collaborative Innovation Center of Genetics and Development, College of Future Technology, Beijing Institute of Genomics, Chinese Academy of Sciences, Beijing 100101, China; g University of Chinese Academy of Sciences, Beijing 100049, China; h Institute of Stem Cell and Regeneration, Chinese Academy of Sciences, Beijing 100101, China; i School of Applied Chemistry and Engineering, University of Science and Technology of China, Hefei 230026, China

**Keywords:** O-GlcNAc, YTHDC1, m^6^A RNA, Homologous recombination, DNA damage repair

## Abstract

N6-methyladenosine (m^6^A) is the most prevalent internal RNA modification, and its regulators include writers, readers and erasers. m^6^A is under stringent control and takes part in many biological events, but it is not known whether there is an interplay between m^6^A and glycosylation. Here we investigated an m^6^A reader, YTHDC1, which has been shown to be recruited to the DNA-RNA hybrid at DNA damage sites and regulate homologous recombination (HR) during DNA damage repair. We found that YTHDC1 is subject to O-linked β-N-acetylglucosamine (O-GlcNAc) modification at Ser396 upon DNA damage, which is pivotal for YTHDC1 chromatin binding and ionization radiation induced focus (IRIF) formation. RNA immunoprecipitation (RIP) and molecular dynamics (MD) simulations indicate that O-GlcNAcylation is vital for YTHDC1 to bind with m^6^A RNA. Fluorescence recovery after photo bleaching (FRAP) analysis revealed that YTHDC1 O-GlcNAcylation is essential for DNA damage-induced YTHDC1-m^6^A condensate formation. We further demonstrate that YTHDC1 O-GlcNAcylation promotes HR-mediated DNA damage repair and cell survival, probably through recruitment of Rad51 to the damage sites. We propose that YTHDC1 O-GlcNAcylation is instrumental for HR.

## Graphical abstract

**Figure:**
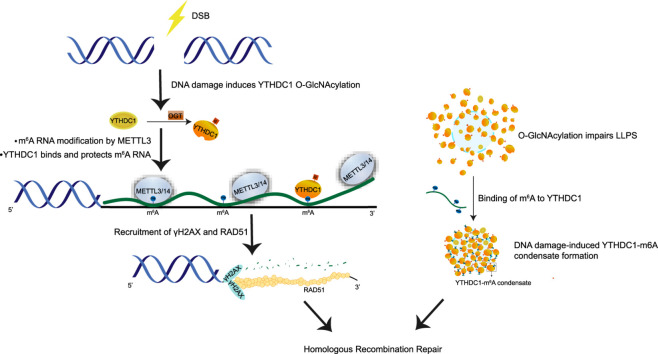

## 1 Introduction

O-linked β-N-acetylglucosamine (O-GlcNAc) is a dynamic glycosylation that occurs intracellularly [1]. It is installed onto the Ser/Thr residues by the only writer O-GlcNAc transferase (OGT), and removed by the sole eraser O-GlcNAcase (OGA) [2]. Currently, this quintessential post-translational modification (PTM) is found to decorate 5000 proteins in the cytosol, nucleus and mitochondria and regulates various biological processes [3].

Emerging evidence suggests that the OGT and OGA pair play an important role in the DNA damage response (DDR) [4]. Genotoxic stimuli from both the environment and our human body induce various types of DNA damage, including DNA double-strand breaks (DSBs) [5,6]. DDR is the countermeasure against these lesions, and in the case of DSB, it is repaired either by nonhomologous end joining (NHEJ) or homologous recombination (HR). NHEJ is more error-prone as it involves the ligation of DSB ends, whereas HR is more precise as its repair templates are the sister chromatids [7,8].

DDR induces protein O-GlcNAcylation, and OGT is recruited to DNA damage sites [9]. O-GlcNAcylation antagonizes DSB-induced phosphorylation of H2AX (γH2AX) to restrict γH2AX to the damage sites [9]. OGA also relocates to DNA damage sites, albeit at a slower kinetics [9], which is mediated by the C-terminus of OGA [10]. Substrates of OGT in DDR have been identified, including mediator of DNA damage checkpoint 1 (MDC1) [9], H2AX [9], and Non-POU Domain Containing Octamer Binding (NONO) [10] and Ku70/80 [10] (the latter two are in the NHEJ pathway). Apart from DSB repair, DNA polymerase eta (Polη) is O-GlcNAcylated during translesion DNA synthesis (TLS), which is a DDR pathway to respond to ultraviolet (UV)- and cisplatin-induced DNA lesions [11]. During DNA synthesis, flap endonuclease 1 (FEN1) is O-GlcNAcylated, and the abrogation of which will lead to DNA damage accumulation [12]. Furthermore, High mobility group B1 (HMGB1) is O-GlcNAcylated, which alters its DNA binding ability and promotes an error-prone DNA repair [13]. Recently, WD repeat and HMG-box DNA-binding protein 1 (WDHD1/AND-1) is also shown to be O-GlcNAcylated and takes part in HR [14]. These data suggest that OGT and its resultant O-GlcNAcylation take part in various DDR pathways and are essential for genome integrity.

Here we demonstrate that O-GlcNAcylation of YT521-B homology-domain-containing protein 1 (YTHDC1) is essential for HR. YTHDC1 is a reader of the N6-methyladenosine (m^6^A) mRNA, which has been identified to be the most prevalent internal mRNA modification since its discovery in 1974 [15,16] . Writers and erasers of m^6^A have been identified, and its readers include the YTH family proteins (YTHDF1-3, and YTHDC1-2) [17]. YTHDC1 has many biological roles [18,19]. In RNA biology, it regulates mRNA splicing by binding with the pre-mRNA splicing factor SRSF3 [20]. The YTHDC1-SRSF3 interaction could be disrupted by the Aurora A-dependent hnRNP K recruitment, resulting in exon skipping [21]. YTHDC1 also promotes the translocation of m^6^A mRNA from the nucleus to the cytosol and facilitates RNA-SRSF3 binding [22]. In liquid-liquid phase separation (LLPS), YTHDC1-m^6^A forms condensates in acute myeloid leukemia (AML) cell lines, and YTHDC1 m^6^A-binding mutants display puncta formation and cell proliferation defects [23]. In addition, m^6^A-modified enhancer RNA (eRNA) also binds with YTHDC1 to form condensates to promote gene activation [24].

YTHDC1 and other m^6^A factors participate in DDR. Upon DSB induction, the m^6^A writer Methyltransferase-like 3 (METTL3) is phosphorylated and relocated to the damage sites, where it mediates m^6^A modification in RNAs involved in DNA damage [25,26]. The resultant m^6^A further recruits YTHDC1 to promote DNA-RNA hybrid accumulation for subsequent HR-mediated DSB repair [26]. *In vitro* studies suggest that the m^6^A writer complex Mettl3-Mettl14 is located at UV-induced cyclopyrimidine dimers to promote correct DDR, and YTHDC1 is also caught at the scene of the crime [27]. In breast cancer cells, Mettl3 promotes EGF expression via m^6^A and resultant YTHDC1 binding, leading to enhanced Rad51 expression and HR [28]. Subsequently, the ablation of Mettl3 impairs HR and leads to Adriamycin (ADR) chemo-sensitivity in breast cancer cells [28]. In AML cells and mouse models, YTHDC1 is essential for not only cell survival and proliferation, but also leukemogenesis, via the essential DNA replication helicase complex component minichromosome maintenance 4 (MCM4) [29].

In this paper, we present evidence that YTHDC1 undergoes O-GlcNAcylated at Ser396 during DNA damage. The glycosylation event promotes YTHDC1 chromatin binding and ionization radiation-induced focus (IRIF) formation. Using RNA immunoprecipitation (RIP) and molecular dynamics (MD) analysis, we show that O-GlcNAcylation promotes DNA damage-induced YTHDC1 condensate formation, probably through YTHDC1-m^6^A binding. We further demonstrate that disrupting the YTHDC1-m^6^A complex leads to defects in γH2AX chromatin dissociation. O-GlcNAcylated YTHDC1 enhances Rad51 recruitment and HR. Collectively, our findings suggest that the OGT-YTHDC1-Rad51 axis promotes HR to protect genome integrity.

## 2 Materials and methods

### 2.1 Cells and antibodies

Cells were grown under standard conditions. The following plasmids were described: YTHDC1 plasmids [20], and OGT plasmids [30]. YTHDC1-S396A was generated following the manufacturers’ instructions (QuickChange II, Stratagene). The primary antibodies were as follows: YTHDC1 (Abcam, ab 122340), γH2AX (Abcam, ab26350), Rad51 (Abcam, ab133534), and m^6^A (Synaptic Systems, 202 003). Chemicals: TMG plus glucose (TMG + Glu) [31] treatment was TMG (5 µM) for 24 h and 30 mM glucose for 3 h; Zeocin: 100 µg/mL for 4 h. siRNA sequences are as follows:

siYTHDC1-1: GTCGACCAGAAGATTATGATA
siYTHDC1-2: ATCGAGTATGCAAATATTGAA

### 2.2 Cell lysis, immunoprecipitation (IP), and Western blotting

The cells were harvested at the indicated time points after relevant treatment and washed twice with phosphate-buffered saline (PBS). The cell pellets were subsequently resuspended in the NETN lysis buffer (20 mM Tris-HCl, pH 8.0, 300 mM NaCl, 1 mM EDTA and 0.5% NP-40). For IB, the lysates were used with indicated antibodies and incubated with Pierce Protein G Agarose (Thermo) for 2–4 h at 4 °C. After washing the agarose beads with NETN100 buffers (20 mM Tris-HCl, pH 8.0, 100 mM NaCl, 1 mM EDTA and 1% NP-40), the samples were mixed with protein loading buffers at 100 °C for 5 min. The protein lysates were separated using 10% SDS‑polyacrylamide gel electrophoresis and equal quantities (5 µg per sample) of separated proteins were transferred to 0.22‑µm nitrocellulose membranes (Millipore Sigma). After blocking with 5% non‑fat milk for 1 h at room temperature, the membranes were incubated with specific primary antibodies overnight at 4 ˚C. After washing three times with PBS, the membranes were incubated with secondary antibodies for 2 h at room temperature. The proteins were visualized using an enhanced chemiluminescence detection kit (Amersham; Cytiva) and the Odyssey Infrared Imaging system version 2.1 (LI‑COR Biosciences). All Western blotting were repeated for at least three times.

### 2.3 DNA damage-induced focus formation assay

Cells were cultured on coverslips and treated with 100 µg/mL Zeocin (a radio-mimetic chemical that induces DSBs) for 4 h. After recovery for 4 h, cells were fixed in 4% paraformaldehyde at room temperature for 15 min, and permeabilized with 0.5% Triton X-100 in PBS for 10 min. Samples were blocked with blocking buffer (8% goat serum in PBS) and then incubated with anti-γH2AX antibodies, followed by fluorescence-labeled secondary antibodies for 1 h. The nuclei were stained with DAPI. The number of foci was counted in at least 100 cells/sample.

### 2.4 RNA immunoprecipitation (RIP) assay

Control and YTHDC1-WT or -S396A overexpressed cells were collected and washed twice with ice-cold PBS. Cells were lysed in ice-cold IP lysis buffers (20 mM Tris-HCl, pH 8.0, 300 mM NaCl, 1 mM EDTA and 0.5% NP-40) for 30 min on ice and spun down at maximum speed to precipitate the debris at 4 °C. Supernatants were collected and incubated with 5 µg anti-YTHDC1 antibodies or an equivalent amount of rabbit IgG by rotating overnight at 4 °C. RNA-YTHDC1-antibody complexes were pulled down using Dynabeads Protein A/G (Millipore) and washed 3 times with NETN100 buffers (20 mM Tris-HCl, pH 8.0, 100 mM NaCl, 1 mM EDTA and 1% NP-40). Proteinase K (Thermo Fisher Scientific Inc.) was applied to remove proteins from the complexes.

### 2.5 Molecular dynamics (MD) simulations

The crystal structure of the YTHDC1 YTH domain with m^6^A RNA was extracted from the complex of RNA GG(m^6^A)CU and YTH (PDB ID: 4R3I) [32]. The O-glycan (*β*-*N*-Acetyl-_D_-Glucosamine) at S396 of YTHDC1 was built using the Glycan Reader & Modeler module [33].

The role of O-glycosylation in YTHDC1 recognition of RNA was investigated via molecular dynamics simulations. Two systems (unglycosylated YTHDC1 and O-GlcNAcylated YTHDC1 at S396 in complex with RNA, respectively) solvated in TIP3P water molecules with 150 mM KCl were built by the CHARMM-GUI webserver [34]. The simulations were performed by the Gromacs 2021.2 program [35,36] with the CHARMM36m force field [37,38]. The systems were equilibrated in the isothermal-isobaric (NPT) ensemble for 200 ns. The SHAKE algorithm was applied to restrain all bonds involving hydrogen [39]. The particle-mesh Ewald (PME) summation method was applied to treat long-range electrostatic interactions [40]. The pressure was set at 1 atm maintained by the Parrinello-Rahman barostat [41] and the temperature was maintained at 310.15 K with the Nosé–Hoover thermostat [42]. Each system was repeated three times independently.

The RMSD (root-mean-square deviation) and RMSF (root-mean-square fluctuation) analyses were performed through MD analysis [43]. The binding energy (enthalpy) and per-residue energy contributions were calculated by the molecular mechanics/Poisson-Boltzmann (generalized-Born) surface area method with the gmx_MMPBSA tool [44,45]. The interactions between YTH and RNA were displayed by PyMol [46].

### 2.6 Fluorescence recovery after photo bleaching (FRAP)

For FRAP experiments in living cells, an area of 1 mm-diameter of YTHDC1 puncta was bleached with a 405 nm laser using an Olympus FV3000 Laser-Scanning Confocal Spectral Inverted Microscope. The GFP fluorescence signal was collected over time. Each data point is representative of the mean and standard deviation of fluorescence intensities in three unbleached (control) or three bleached (experimental) granules. The pre-bleached fluorescence intensity was normalized to 1 and the signal after bleach was normalized to the prebleach level.

### 2.7 Homologous recombination (HR)/non-homologous end joining (NHEJ) *in vivo* reporter assays

DR-GFP-U2OS cells and EJ5-GFP-U2OS cells were overexpressed with YTHDC1-WT or YTHDC1-S396A, and then transfected with JS-20 (SceI) or empty vectors. After 48 h, 2 × 10^5^ cells were analyzed for GFP-positive cells by flow cytometry to demonstrate the repair efficiency of HR and NHEJ. In flow cytometry figures, the y-axis is side scattered light (SSC) to determine the granularity of cells. The x-axis is the GFP signal intensity of cells. GFP-positive populations were gated to distinguish a group of cells with positive signals. Uninduced cells were included as negative controls. The results represent the mean value of triplicate experiments.

### 2.8 Comet assays

To evaluate DNA DSBs, single-cell gel electrophoretic comet assays were performed under neutral conditions. HCT116 cells were overexpressed with YTHDC1-WT or YTHDC1-S396A plasmids with or without 100 µg/mL Zeocin. After incubating in fresh medium for the indicated time at 37 °C, the cells were harvested and mixed with 1% LMP agarose at 42 °C and immediately pipetted onto slides. To perform neutral conditional assays, the slides were immersed in the neutral lysis buffer (2% sarkosyl, 0.5 M EDTA, 0.5 mg/mL proteinase K, pH 8.0) overnight at 4 °C. After lysis, the slides were washed with the electrophoresis buffer (90 mM Tris-HCl at pH 8.5, 90 mM boric acid, 2 mM EDTA), and analyzed by electrophoresis at 25 V for 40 min (0.6 V/cm) and stained in 10 µg/mL propidium iodide for 30 min in the dark. All images were taken under a fluorescence microscope and analyzed by the Comet Assay IV software program.

## 3 Results

### 3.1 DNA damage induces YTHDC1 O-GlcNAcylation at Ser396

**Fig. 1:**
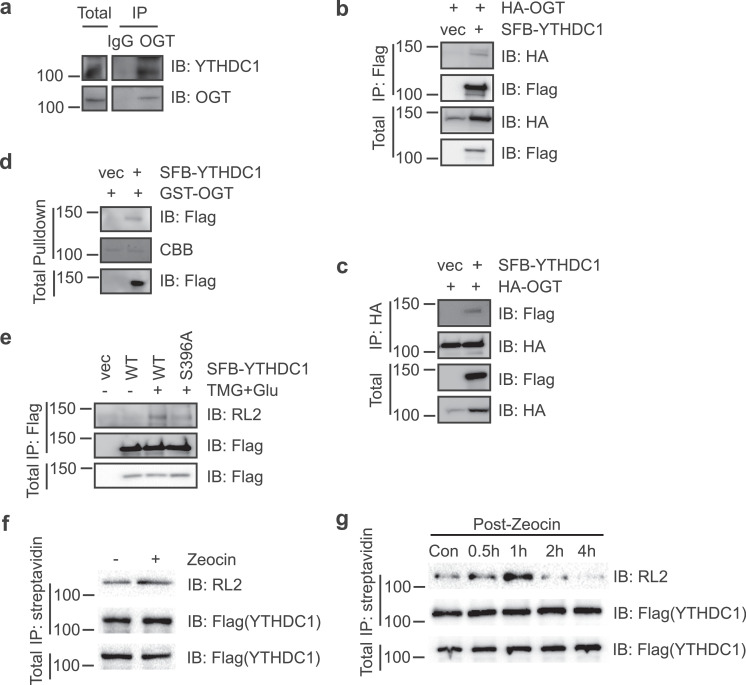
**YTHDC1 is O-GlcNAcylated at S396 upon DNA damage.** (a) Endogenous OGT and YTHDC1 coIP. 293T cellular lysates were immunoprecipitated with anti-OGT antibodies and immunoblotted with anti-YTHDC1 antibodies. (b) Cells were transfected with HA-OGT and SFB-YTHDC1 plasmids. The cellular lysates were immunoprecipitated with anti-Flag (b) or anti-HA (c) antibodies and immunoblotted with the antibodies indicated. (d) Recombinant GST-OGT proteins could pulldown YTHDC1. Cells were transfected with SFB-YTHDC1 or vector controls. The cellular lysates were incubated with GST-OGT proteins purified from *E. coli.* (e) Cells were transfected with YTHDC1-WT, or -S396A plasmids, treated or untreated with TMG plus glucose [31]. The anti-Flag immunoprecipitates were subject to immunoprecipitation and immunoblotting with the antibodies indicated. (f) Zeocin (DNA damage agent) induces O-GlcNAcylation of YTHDC1. HCT116 cells were exposed to the DNA damaging agent Zeocin or mock treatment. The chromatin fractions were extracted and then incubated with streptavidin-conjugated beads at 4°C for 2 hours. The bound protein was subjected to SDS-PAGE and Western blotting with an anti-O-GlcNAc antibody (RL2). (g) DNA damage induces O-GlcNAcylation of YTHDC1 in a time course assay. HCT116 cells were exposed to 100 µg/ml of Zeocin for 4 hours and recovered for different time durations. The cell lysates were examined by Western blotting with RL2 in a time course assay.

Although O-GlcNAcylation has been shown to participate in DDR and increases in response to DNA damage [9], the identification of its substrates has been challenging due to its low stoichiometry, high lability and technical difficulties encountered in mass spectrometry (MS) analysis [2]. Recently a triaryl phosphine-trimethylpiperidine (TFT) reagent was developed to enable one-step identification of ionizing radiation (IR)-induced OGT substrates, and one of the proteins identified is YTHDC1 [47]. Therefore, we first examined the interaction between OGT and YTHDC1. When endogenous interaction was examined, YTHDC1 proteins co-immunoprecipitated (coIP) with OGT (Fig. 1a). We transfected cells with HA-OGT and SFB-YTHDC1, and the cell lysates were immunoprecipitated (IPed) with anti-HA antibodies. SFB-YTHDC1 was found to be in the immunoprecipitates (Fig. 1b). Reciprocally, OGT was also found in the SFB-YTHDC1 immunoprecipitates (Fig. 1c). We then used pull-down assays, in which cells were transfected with SFB-YTHDC1, and the cell lysates were incubated with recombinant GST-OGT proteins. GST-OGT could pulldown SFB-YTHDC1 (Fig. 1d). These results suggest that YTHDC1 and OGT form a complex.

As the TFT-based method suggests that IR-induced O-GlcNAcylation occurs at Ser396, we transfected Flag-YTHDC1 and enriched the lysates for DNA damage-induced O-GlcNAcylation. Then the immunoprecipitates were subject to MS analysis (Table S1), and the results suggest O-GlcNAcylation at Ser396. We then generated S396A mutants of YTHDC1 and examined them for O-GlcNAcylation (Fig. 1e). We used Thiamet-G (TMG, an OGA inhibitor) and glucose incubation to enhance O-GlcNAcylation signals [31]. S396A significantly diminished O-GlcNAcylation levels, suggesting that this is the primary glycosylation site. We also assessed whether the modification responds to DNA damage by Zeocin treatment. As shown in Fig. 1f, Zeocin incubation discernably increased YTHDC1 O-GlcNAcylation signals. When we monitored a Zeocin treatment time course (Fig. 1g), the O-GlcNAcylation of YTHDC1 peaked at approximately 5 hours, and then slowly started to decrease, indicative of a dynamic process of glycosylation.

### 3.2 YTHDC1 O-GlcNAcylation promotes chromatin binding and IR-induced focus (IRIF) formation

**Fig. 2:**
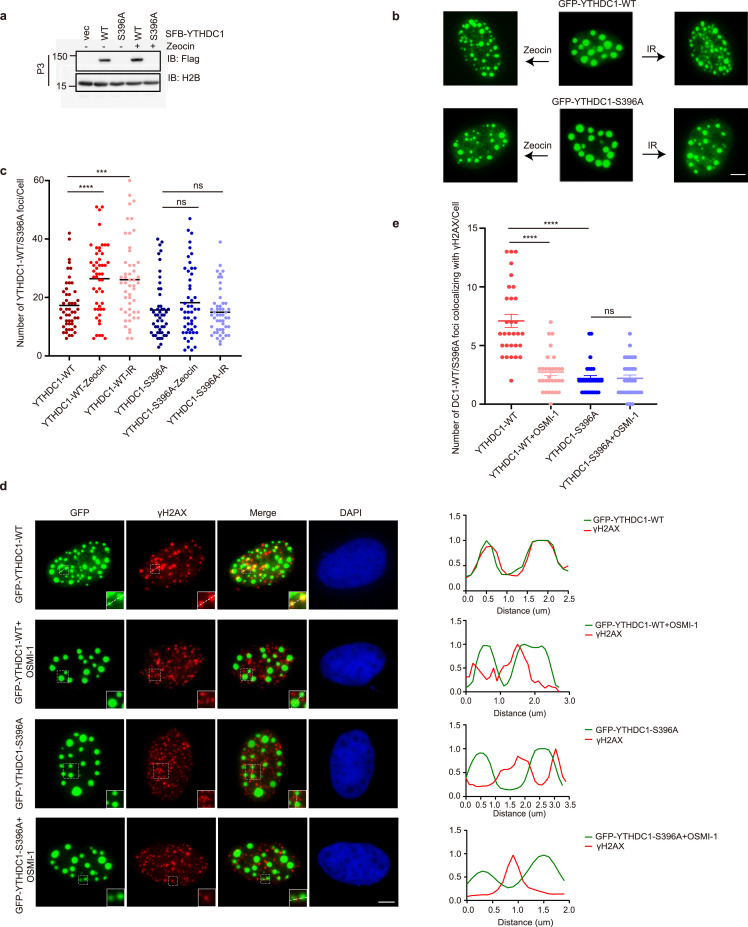
**YTHDC1-S396A is defective in chromatin binding and ionizing radiation-induced focus (IRIF) formation.** (a) 293T cells were transfected with YTHDC1-WT or S396A plasmids and treated with Zeocin. The lysates were then subject to chromatin fractionation assays. The chromatin bound (P3) fraction was immunoblotted with anti-Flag antibodies to detect YTHDC1. (b) U2OS cells were transfected with GFP-YTHDC1-WT, or -S396A plasmids, and then treated with 100 ug/ml Zeocin for 4 hours or 10 Gy X-ray. The GFP foci were quantitated in (c). Scale bar, 5 µm. 45 cells were counted in each experiment. ns: not significant; *** indicates *P*< 0.001, **** indicates *P* < 0.0001. (d) U2OS cells were transfected with GFP-YTHDC1-WT or -S396A plasmids, then treated with 10 Gy X-ray. The OGT inhibitor OSMI-1 was also utilized. The cells were then stained with anti-γH2AX antibodies, and the number of colocalization foci were quantitated in (e). Scale bar, 5 µm.**** indicates *P* < 0.0001.

As YTHDC1 is recruited to DNA damage sites by m^6^A [26], we assessed the chromatin binding of YTHDC1-S396A mutants. As shown in Fig. 2a, YTHDC1-WT and -S396A mutants were transfected into cells and chromatin fractionation was carried out. We also treated the cells with Zeocin. As a result, S396A abolished the chromatin binding of YTHDC1. We also constructed GFP-YTHDC1 to visualize YTHDC1 localization in living cells. Under basal conditions, GFP-YTHDC1-WT and -S396A both formed foci. Upon Zeocin treatment or IR, WT cells showed a robust increase of focus formation, while the S396A mutant was still at the basal level (Fig. 2b-c).

We then examined γH2AX induction by cytology as it is a marker for DSB [26]. In WT cells, Zeocin treatment significantly induced γH2AX localization, with a good correlation with GFP-YTHDC1 signals (Fig. 2d-e), consistent with previous observations [26]. In S396A cells, however, γH2AX intensity correlated poorly with YTHDC1 (Fig. 2d-e), suggesting that YTHDC1 O-GlcNAcylation is essential for its relocation to the damage sites. To exclude the possibility that these effects were caused by the mutation of S396A, we also utilized the OGT inhibitor OSMI-1 in these experiments (Fig. 2d-e and S1). Upon OGT inhibition, the cells displayed the same phenotype as the S396A mutant, suggesting that O-GlcNAcylation is indeed a prerequisite for YTHDF1 recruitment to the damage sites.

### 3.3 YTHDC1 O-GlcNAcylation enhances m^6^A binding

**Fig. 3:**
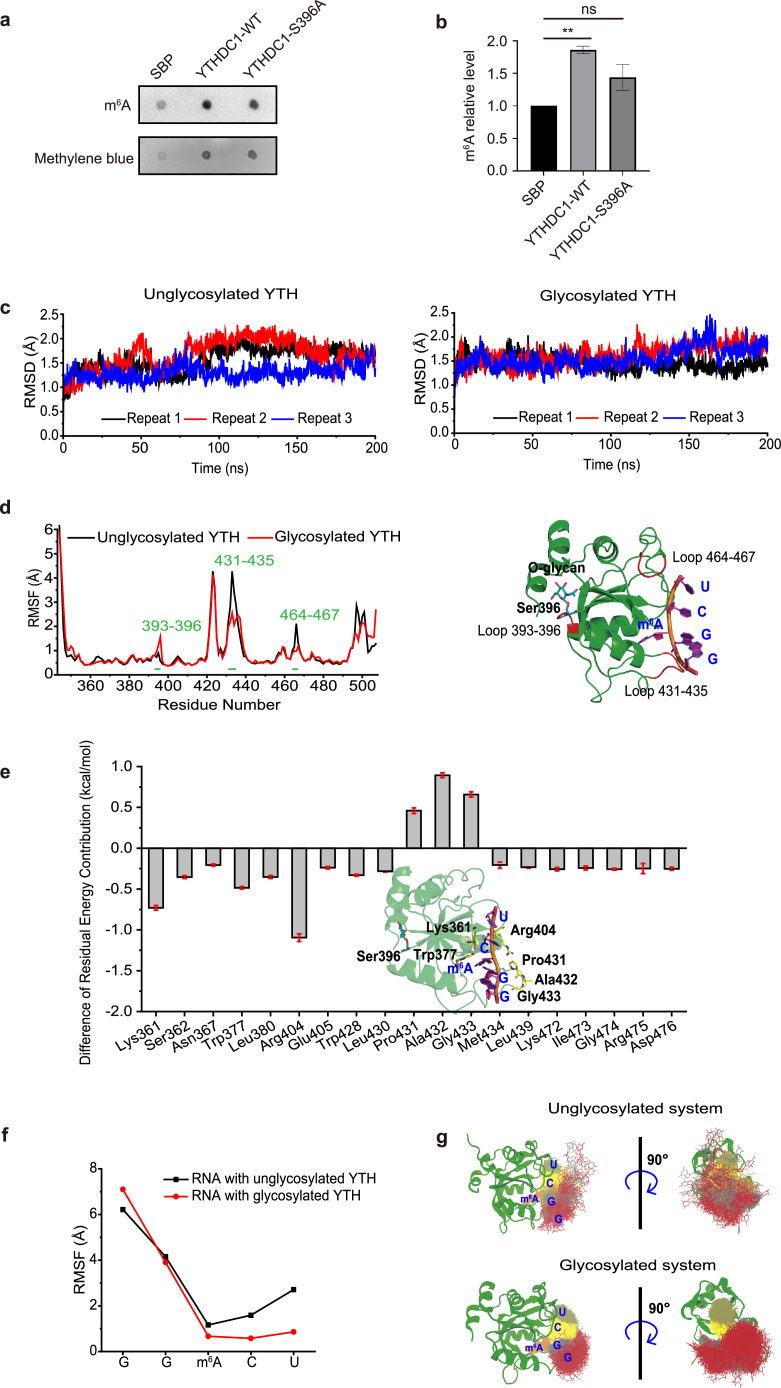
**YTHDC1-S396A is defective in m^6^A mRNA binding.** (a) RNA Immunoprecipitation (RIP) assay showed that YTHDC1-S396A is defective in m^6^A mRNA binding. (b) Quantitation of (a). ns indicates not significant, ** indicates *P* < 0.01. (c-g) Molecular dynamics simulations indicate that glycosylated YTHDC1 reduced m^6^A mRNA binding through an allosteric regulation. (c) Root-mean-square deviations (RMSDs) of Cα atoms of unglycosylated YTHDC1 (left) and glycosylated YTHDC1 (right) with RNA in the independent repeated simulations (Repeat 1, black; Repeat 2, red; and Repeat 3, blue). (d) Root mean square fluctuations (RMSFs) of the Cα atoms of unglycosylated (black) and glycosylated (red) YTHDC1 in the combined 240 ns trajectories. (e) Differences in each residue energy contribution between the unglycosylated system and the glycosylated system (only the residues with contributions > 0.2 kcal/mol are shown). The inset shows a schematic view of the key residues around RNA. (f) RMSF of bases in unglycosylated (black) and glycosylated (red) YTHDC1 in the combined 240 ns trajectories. (g) Overlay of RNA structures from the combined 240 ns trajectories.

As YTHDC1 is recruited to the damage sites via m^6^A binding [26], we tested the m^6^A binding ability of YTHDC1 via RNA immunoprecipitation (RIP) assays followed by dot blot (Fig. 3a-b). The S396A mutant significantly decreased m^6^A binding (Fig. 3a-b), which could be part of the reason for YTHDC1 localization defects during DDR.

Aiming to probe the potential modulatory role of O-GlcNAc on Ser396 at the molecular level, we first attempted to synthesize the O-GlcNAcylated YTH domain of YTHDC1 via a chemical approach, which should afford a homogeneous glycoprotein that would be inaccessible through bioexpression protocols. Accordingly, we dissected the YTH domain into four segments 1a-4a that could be prepared using solid-phase peptide synthesis and were subsequently assembled based on chemical ligation and desulfurization strategies (Supplementary materials). While the peptidyl hydrazide-based ligations proceeded efficiently, we noticed that the desulfurization steps were problematic, either leading to incomplete conversion of intermediates, such as peptide 6a, or generating insoluble aggregates at the final stage of assembling full length protein 10a. Eventually, we managed to obtain a small amount of the S396-GlcNAcylated YTH domain (11a). The synthetic protein displays an analogous CD spectrum to that of the nonglycosylated YTH domain obtained from *E. coli* expression (Supplementary materials), suggesting comparable secondary structures. Unfortunately, the synthetic route was unable to provide sufficient materials for further biochemical and biological evaluations.

To circumvent the difficulties of accessing S396-GlcNAcylated YTHDC1, we resorted molecular dynamics (MD) simulation. The root-mean-square deviation (RMSD) values were first calculated. As shown in Fig. 3c, both systems can reach stable states in 200 ns. The frames of the last 80 ns were extracted from the trajectory of each repeat and combined together for further analysis for each system (240 ns in total). To verify whether O-GlcNAc influences the fluctuation of each residue during simulations, the root-mean-square fluctuations (RMSFs) were calculated for the combined 240 ns trajectories. The existence of O-GlcNAc disturbed the loop (Residues 393-396) where it is located, but decreased the fluctuation of the loop close to the RNA binding site (Residues 431-435 and 464-467) (Fig 3d). The binding energy of glycosylated YTH to RNA was -85.96 ± 0.18 kcal/mol, which is lower than that of unglycosylated YTH to RNA (-74.86 ± 0.51 kcal/mol). The differences in each residue contribution between the unglycosylated system and the glycosylated system are shown in Fig. 3e. After S396 was O-GlcNAcylated, RNA GG(m^6^A)CU was further stabilized by Lys361 and Arg404 with electrostatic interactions.

The behavior of RNA in the binding site was also investigated. The methylated adenosine (m^6^A) was deeply inserted into the binding site and was not influenced by O-glycosylation (Fig 3f). Regardless of whether S396 was O-GlcNAcylated, the GG motif of RNA was equally flexible. However, glycosylation dramatically restricted the fluctuation of the CU motif of RNA (Fig. 3g). Overall, the glycosylation increased the binding affinity of RNA to the YTH domain and stabilized the binding of RNA, probably through an allosteric regulation.

### 3.4 YTHDC1 O-GlcNAcylation facilitates DNA damage-induced YTHDC1-m^6^A condensate formation

**Fig. 4:**
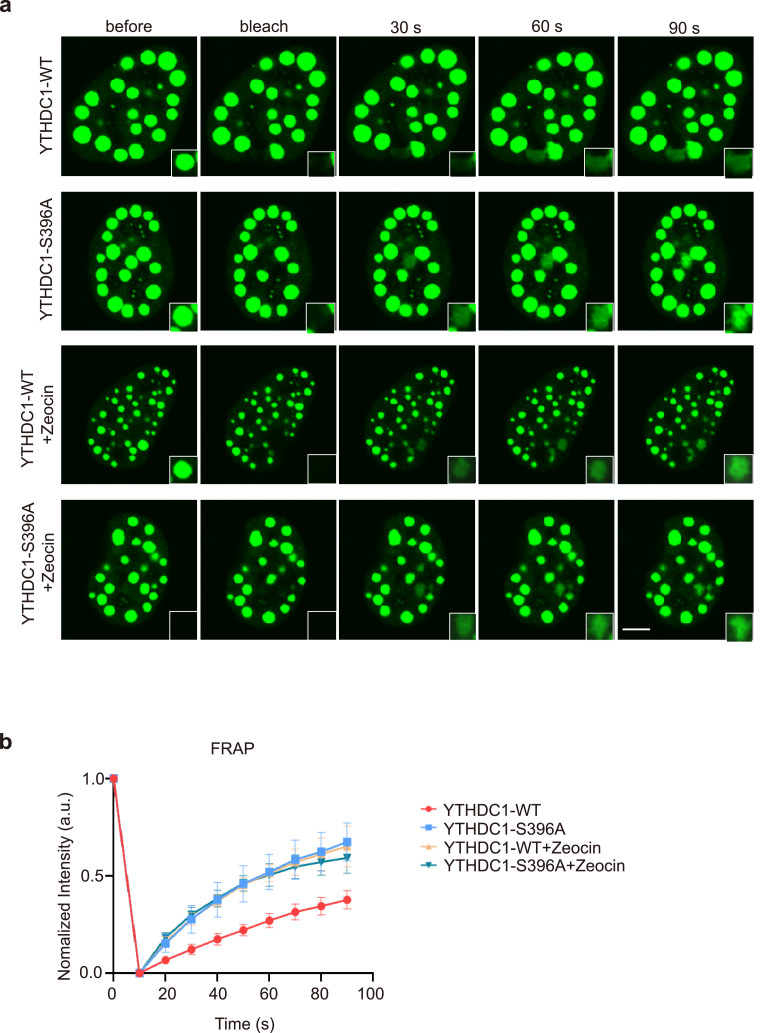
**YTHDC1 O-GlcNAcylation enhances YTHDC1-m^6^A condensate formation upon DNA damage.** (a) Fluorescence recovery after photo bleaching (FRAP) of GFP-YTHDC1-WT and -S396A droplets *in vivo*, with or without Zeocin treatment. Scale bar, 5 uM. (b) Quantitation of the results in (a) (N = 3).

Recent studies have shown that O-GlcNAcylation inhibits LLPS [48,49], and YTHDC1-m^6^A is implicated in LLPS [23,24], we therefore examined how YTHDC1 affects condensate formation. As shown in Fig 4a-b, fluorescence recovery after photo bleaching (FRAP) assays showed that under basal conditions, O-GlcNAcylation opposes LLPS, as indicated by the faster recovery rate and higher intensity of GFP-YTHDC1-S396A compared to WT (Fig. 4a-b).

Upon Zeocin treatment, however, YTHDC1-WT and S396A responded completely differently. YTHDC1-WT manifested much more DNA damage-induced foci, consistent with our previous results (Fig. 2b). Moreover, Zeocin treatment increased the LLPS rate in YTHDC1-WT-transfected cells, which is in line with the recent reports that m^6^A enhances the phase separation of many m^6^A reader proteins [50], [51], [52], including YTHDC1 [23]. On the other hand, the FRAP rate in YTHDC1-S396A remained the same as that in untreated cells, suggesting that DNA damage-induced O-GlcNAcylation could account for the difference.

### 3.5 YTHDC1 O-GlcNAcylation promotes HR

**Fig. 5:**
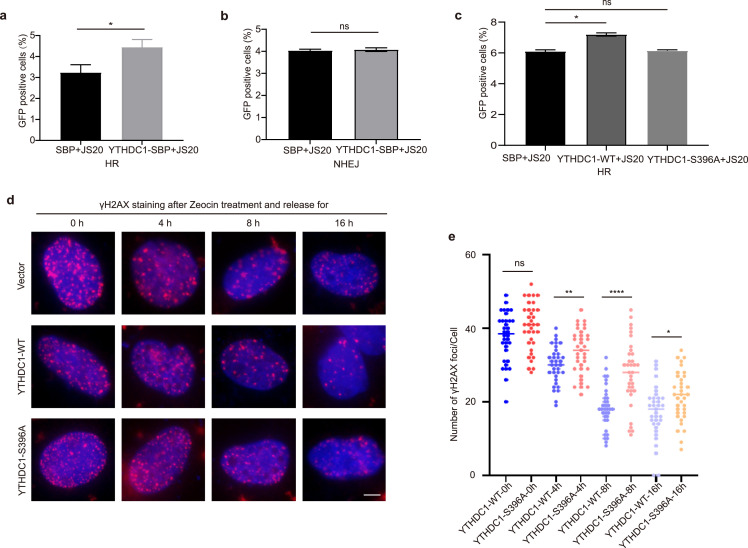
**YTHDC1 O-GlcNAcylation promotes DSB repair.** (a) DR-U2OS cells were infected with I-SceI and YTHDC1-WT or control plasmids to detect homologous recombination (HR). The percentage of GFP-positive cells is shown. YTHDC1-WT transfected cells showed more HR-mediated DSB repair. * indicates *P* < 0.05. (b) EJ5-GFP 293 cells were transfected with I-SceI and YTHDC1-WT or control plasmids to detect non-homologous end joining (NHEJ). The percentage of GFP-positive cells is shown. There is no significant difference between YTHDC1-WT and control cells. NS: not significant. (c) DR-U2OS cells were infected with I-SceI and YTHDC1-WT or -S396A plasmids. HR-mediated DSB repair was measured by flow cytometry. NS: not significant. * indicates *P* < 0.05. (d-e) YTHDC1-S396A cells displayed defects in γH2AX dissociation. Cells were transfected with GFP-YTHDC1-WT, or -S396A plasmids, treated with Zeocin and released for the time indicated. Then the cells were stained with anti-γH2AX antibodies and quantitated (e). Scale bar, 5 µm. ns: not significant; * indicates *P* < 0.05, ** indicates *P* < 0.01, **** indicates *P* < 0.0001.

Since the data above suggest that YTHDC1 O-GlcNAcylation plays a role in regulating DDR, we sought to determine whether it regulates HR or NHEJ. Using the established GFP reporter assays [53], we found that YTHDC1 modulates HR (Fig. 5a), but not NHEJ (Fig. 5b), consistent with published results [26,28]. We then transfected YTHDC1-S396A into the DR-GFP system to monitor HR, and the results showed decreased HR efficiency (Fig. 5c), suggesting that S396 O-GlcNAcylation is essential for HR.

We next analyzed γH2AX dissociation after Zeocin treatment, as it is an indication of DDR completion [54]. As shown in Fig. 5d, γH2AX foci started to decrease rapidly 4 hours after release from Zeocin treatment and continued to decline until 16 hours. In contrast, in the S396A cells, γH2AX foci remained much more abundant compared to the WT. This is consistent with an HR defect in the S396A mutant.

### 3.6 YTHDC1 O-GlcNAcylation promotes HR through Rad51 recruitment

**Fig. 6:**
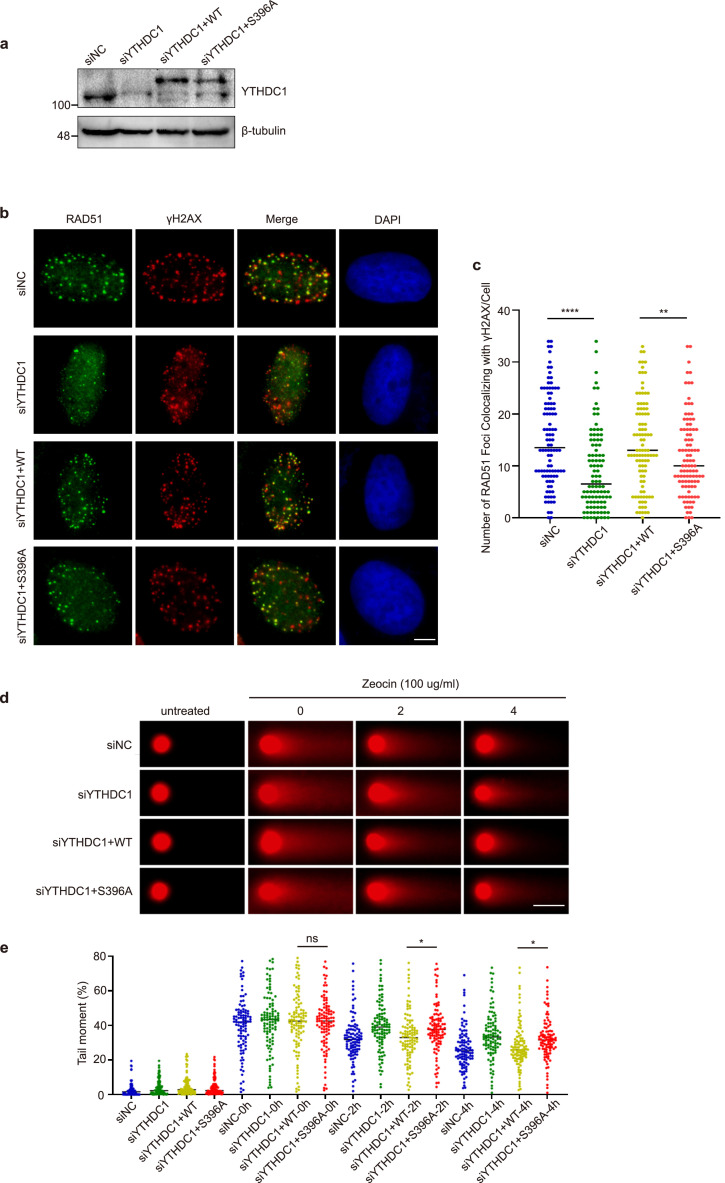
**YTHDC1-WT modulates HR through Rad51 recruitment.** (a) U2OS cells were treated with siRNA targeting YTHDC1 and then transfected with YTHDC1-WT or S396A plasmids. (b) The cells in (a) were treated with Zeocin, and co-stained with anti-Rad51 and anti-γH2AX antibodies. Scale bar, 5 µm (c) Quantitation of the co-localization between Rad51 and γH2AX. ** indicates *P* < 0.01, **** indicates *P* < 0.0001. (d) YTHDC1-S396A is defective in DNA damage repair as analyzed by neutral comet assays. The cells in (a) were treated with Zeocin and then recovered for the time indicated. Scale bar, 50 µm. Comet tails were analyzed, and the DNA damage repair kinetics were assessed (e). ns indicates not significant, * indicates *P* < 0.05.

Then we sought to identify the downstream targets of YTHDC1. The Rad51 recombinase plays a crucial role in HR, as it assembles into helical filament wrapping the single-strand DNA tails at the DSBs. Its recruitment and accumulation at the DSBs are usually used as a marker for HR [5,6]. We first depleted endogenous YTHDC1 with siRNA and then transfected YTHDC1-WT and -S396A (Fig. 6a). We measured Rad51 recruitment using these cells because the METTL3-m^6^A-YTHDC1 axis is implicated in Rad51 recruitment [26]. As shown in Fig. 6b-c, YTHDC1 depletion significantly reduced Rad51 focus formation, in line with previous findings that YTHDC1 upregulates Rad51 expression [28]. Compared with YTHDC1-WT, S396A significantly attenuated Rad51 focus formation (Fig. 6b-c), suggesting that YTHDC1 O-GlcNAcylation is essential for Rad51 localization to DSB sites.

**Fig. 7:**
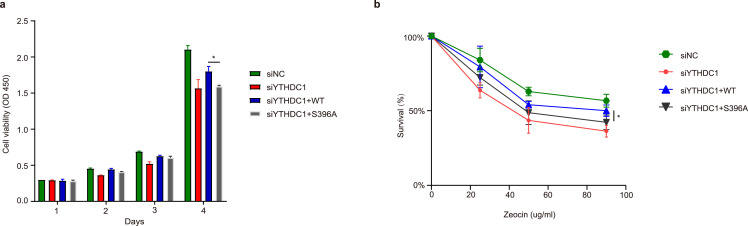
**YTHDC1 O-GlcNAcylation is essential for cell survival.** The cells were treated with Zeocin for different durations or different doses, and CCK-8 assays were performed in the indicated cell lines. * indicates *P* < 0.05.

Then we measured for DSB repair kinetics by comet assays using the same cells [10]. Zeocin was used to induce DSBs, and knockdown of YTHDC1 or reconstitution of S396A significantly impaired DSB repair (Fig. 6d-e). Furthermore, we used different doses of Zeocin to treat the cells and carried out CCK-8 assays (Fig. 7a-b). Once again, the S396A mutant displayed hypersensitivity. Collectively, these results suggest that YTHDC1 O-GlcNAcylation is essential for DDR, perhaps through Rad51 recruitment.

## 4 Discussion and conclusion

In this work, we present evidence that YTHDC1, an m^6^A reader, undergoes O-GlcNAcylation upon DNA damage, which is indispensable for its chromatin binding, m^6^A mRNA binding and subsequent recruitment to DNA damage sites. Through biochemical and cytological analysis, we show that YTHDC1 O-GlcNAcylation promotes γH2AX recruitment and Rad51 localization and enhances DDR through HR. As recent studies have shown that small RNAs are N-glycosylated on the cell surface [55], our findings reveal that the m^6^A mRNA reader YTHDC1 is O-GlcNAcylated in the cell, suggesting a potential interconnection between RNA and glycosylation.

Among the OGT substrates that have been identified, O-GlcNAcylated sites often reside in intrinsically disordered regions (IDRs). In contrast, YTHDC1 O-GlcNAcylation occurs at S396, which is located right in the YTH domain, the m^6^A binding domain. Through RIP assays and MD simulations, we show that S396 O-GlcNAcylation is important for YTHDC1- m^6^A binding. We propose that there might be other O-GlcNAcylated sites on functional domains in addition to IDRs.

Recently, the relationship between O-GlcNAc and LLPS has been the subject of intense investigation. For instance, Thr1306 O-GlcNAcylation of neuronal SynGAP has been demonstrated to inhibit the formation of the SynGAP-PSD-95 complex and thus LLPS [49]. In addition, O-GlcNAc attenuates LLPS of the RNA-binding EWS N-terminal disordered region [48]. In our study, we found that under basal conditions, O-GlcNAcylation inhibited YTHDC1 LLPS (Fig. 4a). However, upon DNA damage, YTHDC1-S396A displayed no significant changes compared to the untreated control. YTHDC1-WT, on the other hand, exhibited phase separation phenotypes, probably due to increased binding with m^6^A and subsequent formation of YTHDC1- m^6^A condensates. These results suggest that the effect of O-GlcNAcylation on LLPS varies depending on the cellular context and that different binding partners may exert various effects on LLPS.

Structure-based MD simulations have gained popularity to facilitate probing the functional properties of glycosylated proteins, probably due to the current challenges in the chemical synthesis of glycopeptides or glycoproteins. Previous studies have used MD simulations to predict the effects of O-GlcNAc glycoengineering of GPCRs, with simulations suggesting that such modifications enhance proteolytic stability [56]. Recent work on malate dehydrogenase 1 (MDH1) using MD simulations indicates that O-GlcNAc strengthens MDH1-substrate binding, probably as a molecular glue [57]. When chemical synthesis does succeed, it no doubt pinpoints the exact role of O-GlcNAc on a given protein or peptide. For instance, a removable glycosylation modification (RGM) method was used in total chemical synthesis, and O-GlcNAc was found to improve disulfide-rich protein folding [58]. In the case of α-synuclein, which is implicated in neurodegenerative diseases, O-GlcNAc inhibits amyloid aggregation [59]. Although we could not synthesize O-GlcNAcylated YTHDC1 protein, our MD simulations suggest that O-GlcNAc could impose an allosteric regulation of YTHDC1, thereby enhancing YTHDC1-m^6^A binding. This represents yet another unappreciated function of O-GlcNAcylation. In the future, perhaps a combination of chemical synthesis and MD simulations would yield even more interesting results, shedding light on the multifaceted roles of O-GlcNAc.

The exact function of OGT in DDR is far from clear. Due to the difficulty in pinpointing OGT modification sites, it is uncertain whether OGT or OGA plays a major role in HR or NHEJ. Both OGT and OGA are recruited to damage sites, although OGA is recruited at a slower rate [9]. One possible scenario that OGT is recruited to the DNA damage sites first to catalyze protein O-GlcNAcylation, and OGA reverses the reaction after the substrates have carried out their function. Another scenario is that these two enzymes catalyze distinct sets of substrates for totally different functions. Reports of O-GlcNAcylated proteins in both pathways have been anecdotal: MDC1(9), H2AX [9], AND-1(14) in HR, and NONO [10] and Ku70/80 [10] in NHEJ. The potential role of O-GlcNAc in other types of damage, such as DNA single-strand break, has not been investigated. Due to the transient nature of DNA damage and the repair process, new methodologies have been developed to identify the O-GlcNAcylated substrates in a time-resolved fashion [12,47]. Perhaps a more concerted role of OGT or OGA in DDR will be unveiled with more chemoproteomic studies in the future.

## Data availability statement

All data are contained in this manuscript.

## Declaration of competing interest

The authors declare that they have no conflicts of interest in this work.

## Appendix Supplementary materials

Image, application 1

Image, application 2

Image, application 3
