# Supplementary material for: DNA damage-induced YTHDC1 O-GlcNAcylation promotes homologous recombination by enhancing m6A binding
Source: Fundam Res. 2023 Jun 5;5(2):868–79. doi: 10.1016/j.fmre.2023.04.017 (PMC11997583; doi:10.1016/j.fmre.2023.04.017)
Supplement: Supplementary file 2 [file mmc2.pptx]

## Slide 1
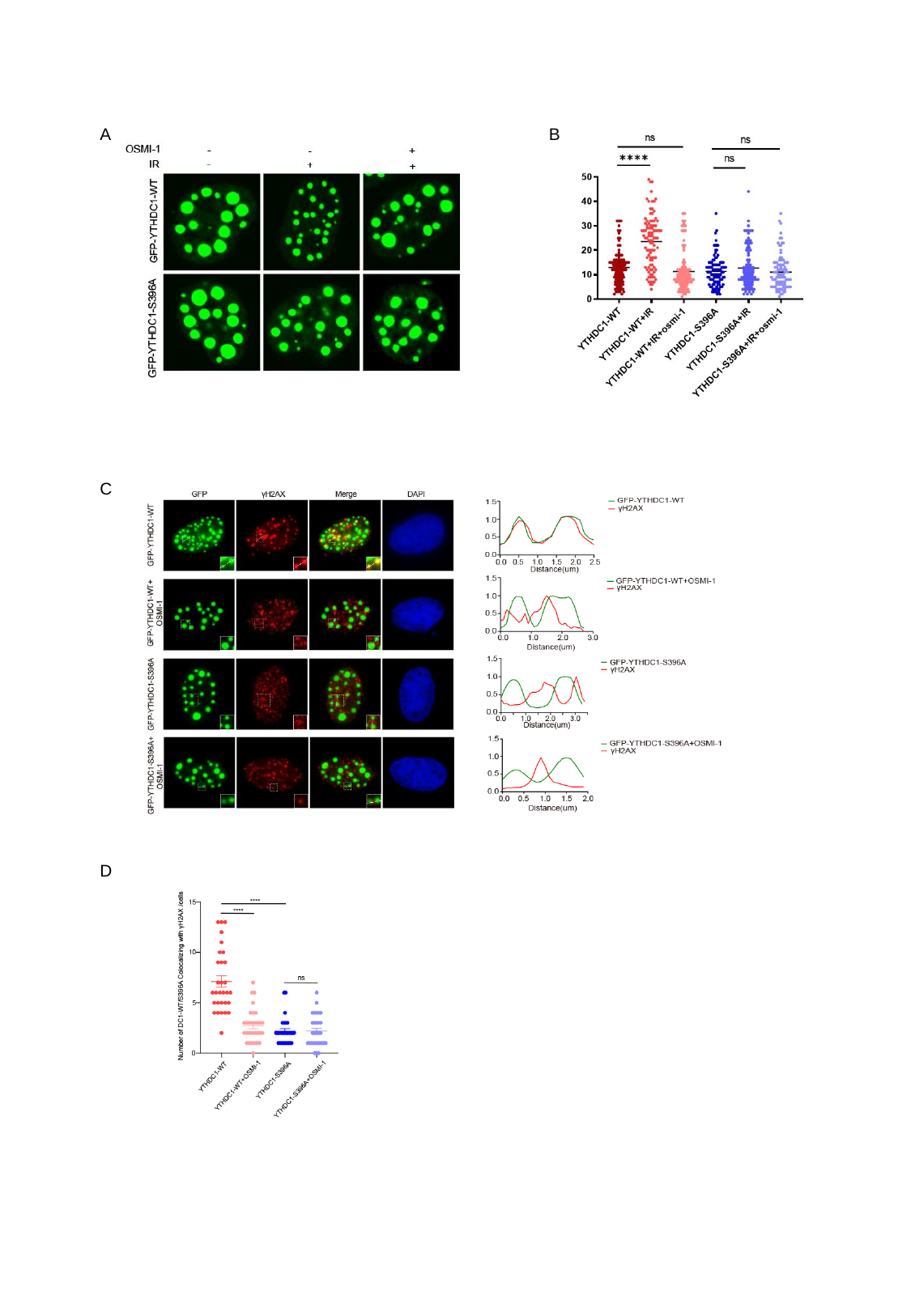

A
B
C
D

## Slide 2
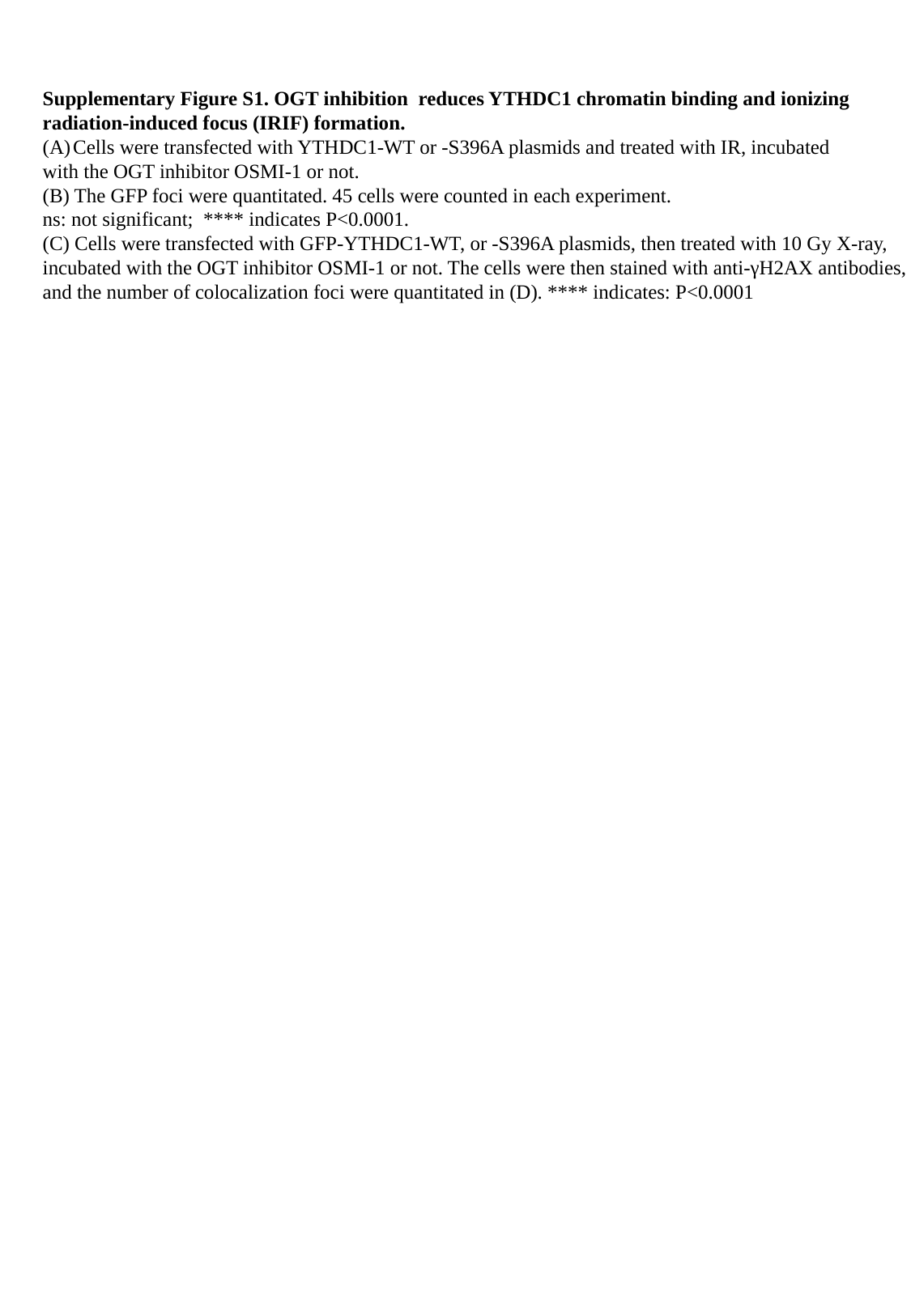

Supplementary Figure S1. OGT inhibition reduces YTHDC1 chromatin binding and ionizing
radiation-induced focus (IRIF) formation.
Cells were transfected with YTHDC1-WT or -S396A plasmids and treated with IR, incubated
with the OGT inhibitor OSMI-1 or not.
(B) The GFP foci were quantitated. 45 cells were counted in each experiment.
ns: not significant; **** indicates P<0.0001.
(C) Cells were transfected with GFP-YTHDC1-WT, or -S396A plasmids, then treated with 10 Gy X-ray,
incubated with the OGT inhibitor OSMI-1 or not. The cells were then stained with anti-γH2AX antibodies,
and the number of colocalization foci were quantitated in (D). **** indicates: P<0.0001
